# Intrathecal Inflammation in Progressive Multiple Sclerosis

**DOI:** 10.3390/ijms21218217

**Published:** 2020-11-03

**Authors:** Salvatore Monaco, Richard Nicholas, Richard Reynolds, Roberta Magliozzi

**Affiliations:** 1 Department of Neurosciences, Biomedicine and Movements Sciences, University of Verona, 37134 Verona, Italy; 2 Department of Brain Sciences, Imperial College, Faculty of Medicine, London W12 ONN, UK; r.nicholas@imperial.ac.uk (R.N.); r.reynolds@imperial.ac.uk (R.R.)

**Keywords:** inflammation, demyelination, neurodegeneration, cytokines, meninges, ependyma, cerebrospinal fluid, choroid plexus, autoimmunity

## Abstract

Progressive forms of multiple sclerosis (MS) are associated with chronic demyelination, axonal loss, neurodegeneration, cortical and deep gray matter damage, and atrophy. These changes are strictly associated with compartmentalized sustained inflammation within the brain parenchyma, the leptomeninges, and the cerebrospinal fluid. In progressive MS, molecular mechanisms underlying active demyelination differ from processes that drive neurodegeneration at cortical and subcortical locations. The widespread pattern of neurodegeneration is consistent with mechanisms associated with the inflammatory molecular load of the cerebrospinal fluid. This is at variance with gray matter demyelination that typically occurs at focal subpial sites, in the proximity of ectopic meningeal lymphoid follicles. Accordingly, it is possible that variations in the extent and location of neurodegeneration may be accounted for by individual differences in CSF flow, and by the composition of soluble inflammatory factors and their clearance. In addition, “double hit” damage may occur at sites allowing a bidirectional exchange between interstitial fluid and CSF, such as the Virchow–Robin spaces and the periventricular ependymal barrier. An important aspect of CSF inflammation and deep gray matter damage in MS involves dysfunction of the blood–cerebrospinal fluid barrier and inflammation in the choroid plexus. Here, we provide a comprehensive review on the role of intrathecal inflammation compartmentalized to CNS and non-neural tissues in progressive MS.

## 1. Introduction

Multiple sclerosis (MS) is a chronic autoimmune disorder characterized by white matter and gray matter demyelination, axonal loss, and neurodegeneration [1]. MS displays heterogeneity in clinical manifestations and outcome, in addition to distinctive clinical courses, including relapsing/remitting MS (RRMS), secondary progressive MS (SPMS), or primary progressive (PPMS) [2]. Whilst acute cellular inflammation with breakdown of the blood–brain barrier (BBB) is observed in RRMS, progressive disease is associated with compartmentalized chronic inflammation at leptomeninges and brain parenchyma, in the presence of an intact BBB [3]. In progressive MS, parenchymal inflammation is sustained by perivascular infiltrates of tissue-resident memory T cells, lesion border macrophages, and activated microglia. Owing to its gradual progression and clinical heterogeneity, the transition from RRMS to SPMS is difficult to define based on clinical features and can result in diagnostic and treatment delay.

Recent research on MS has provided insights into pathogenetic mechanisms governing the progressive forms of the disease, which are characterized by increasing disability, neurodegeneration, and gray matter atrophy. These changes are thought to be the effect of mechanisms triggered by compartmentalized chronic innate and adaptive immune responses in the brain parenchyma and CSF-filled regions and associated cells lining these areas [4]. The latter include the meninges and the cerebrospinal fluid (CSF), which is in part secreted by epithelial cells of the choroid plexuses (CP) of the brain’s ventricles, and the remaining half contributed by the interstitial fluid of the brain and by the secretory epithelium of ependymal cells lining the cerebral ventricles [5,6].

At different interfaces between the blood–CNS and blood–non-neural tissues, including meningeal membranes and choroid plexuses, blood vessels display distinctive cellular properties and biochemical mechanisms that provide the basis for anatomical and immunological barriers. These barriers, encompassing the BBB, the blood–meningeal barrier (BMB), and the blood–CSF (B-CSF) barrier, regulate the active transport of small molecules while restricting the inward passage of cells and large molecules [7]. Additionally, non-vascular barriers are provided by the epithelial arachnoid and pial layers at the interface between the subarachnoid CSF and dura and pia mater and by the ependymal barrier of glial cells exposed to ventricular CSF and brain interstitial fluid. Among BBB, B–CSF, and BMB, immune cell entry into CNS and CSF occurs only through the endothelium of the cerebrovasculature and the choroid plexus epithelium [8]. The precise mechanisms involved in compartmentalization of cellular inflammation to distinctive intrathecal niches, such as meninges and CP, and their correlation with damage to neighboring nervous tissues remain to be fully clarified. Over the last decade, insight into pathogenetic mechanisms of MS has been gained from neuropathological studies of post-mortem tissues and molecular proteomic analysis of blood/CSF [9,10], and this review describes current knowledge about the role of intrathecal inflammation compartmentalized in the CSF and non-neural tissues.

## 2. Meningeal Inflammation

**Figure 1:**
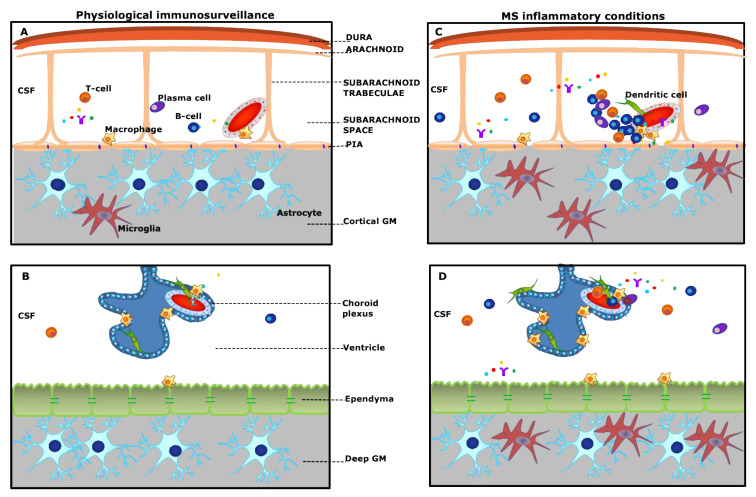
Schematic overview of “meningeal and choroid plexus immunity” (**A**,**B**) and pathological alterations in multiple sclerosis (MS) (**C**,**D**). (**A**): The meninges consist of different membranes, including the dura mater (periosteal and meningeal layers), the arachnoid, and the pia. Strands of connective tissue, or arachnoid trabeculae, extend from the arachnoid layer and contains the vascular system. Under physiological conditions, the subarachnoid space is populated by scattered B- and T-cells, plasma cells, and macrophages and is filled with the cerebrospinal fluid (CSF) bathing the cortical grey matter (GM). (**C**): In MS, either innate or adaptive immune cells, in particular B-cells, accumulate in the subarachnoid space, outside the meningeal vessel, and may form ectopic tertiary lymphoid-like structures (TLSs), which are in close contact with the pia mater. This compartmentalized meningeal inflammation may contribute to the release in the CSF of inflammatory mediators that diffuse through the pia and induce subpial cortical demyelination. (**B**): The choroid plexus (CP) stroma is irrigated by fenestrated microvessels allowing free diffusion of blood-borne molecules. The CP epithelium surrounds the stroma and forms the blood–CSF barrier. Under physiological conditions, dendritic cells, macrophages, and sparse T-cells are found in the choroid plexus stroma. (**D**): During chronic neuroinflammation, both innate and adaptive immune cells accumulate in the choroid plexus and release inflammatory and proinflammatory factors in the surrounding CSF. These inflammatory changes may possibly induce changes in the structure and function of the ependyma lining the ventricle and the sub-ependymal glia, similar to those observed in the subpial GM.

Intracranial and intraspinal structures that wrap the brain include the three layers of meninges consisting of the dura mater; the arachnoid mater, which is filled with the cerebrospinal fluid and contains leptomeningeal vessels; and the densely vascularized pia mater [11,12]. The dural and subdural meninges are mostly populated with macrophages, and to a lesser extent with resident and circulating T cells, in addition to B cells, dendritic cells, and natural killer cells. These populations, including cells of the innate and adaptive divisions and innate lymphoid elements, which incorporate features of both innate and adaptive divisions, are collectively referred to as “meningeal immunity” and are involved in meningeal immune surveillance and in exerting effector functions [13]. Beside the heterogeneity in immune cellular composition, which is more complex within dural layers, meningeal membranes are characterized by the presence of the arachnoid barrier (AB) cells at the interface with the dura and the blood–meningeal barrier (Figure 1A,B). While the AB is considered an insurmountable hurdle for the inward passage to the CSF of molecules released from leaky dural capillaries, and, in addition, no evidence exists for the involvement of the dura mater in MS, the MRI detection of dural nodular enhancement, close to veins and sinuses [14], remains to be elucidated.

Over the last 15 years, intrathecal accumulation of B cells forming ectopic tertiary lymphoid-like structures (TLSs), morphologically resembling B-cell follicles of secondary lymphoid follicles in severe cases, has been demonstrated within the inflamed meninges of a subgroup of MS patients with more rapid and severe disease progression [15,16,17,18] as well as in brain biopsies obtained from patients with early stage MS [19] (Figure 1C). Similar ectopic TLSs have been previously detected also in inflamed tissues in autoimmune conditions, such as rheumatoid arthritis, myasthenia gravis, Hashimoto’s thyroiditis, and Sjögren’s syndrome; other chronic inflammatory diseases, including ulcerative colitis and Chron’s diseases; infectious diseases—namely, chronic hepatitis C, and chronic Lyme disease—in addition to many tumor types, and were considered as sites of chronic local inflammation in the setting of systemic inflammatory diseases [20]. The degree of TLS organization correlates with tissue infiltration, indicating that induction of robust tertiary lymphoid organs (TLOs) largely depends on the magnitude of local immune activation. Moreover, it has been suggested that extant TLSs may engender local adaptive immune responses toward locally displayed antigens, resulting in turn in a more aggressive disease with a poor prognosis, autoantibody production, and increased risk for the development of malignancies in many non-neural tissues [21]. Typically, TLSs develop during the transition from acute to chronic inflammation and are accompanied by the ectopic expression of lymphoid chemokines CCL19, CCL21, CXCL13, and CXCL12. In particular, CXCL13 is supplied by the predominant population of stromal cells in the B-cell follicle, the follicular dendritic cells (FDCs) [20]. The presence of meningeal TLS in cerebral sulci of a subgroup of post-mortem MS cases (accounting for about 40% of examined cohorts) correlated with the extent of subpial cortical demyelination, also named type III cortical lesions, and more severe and rapid disease progression, characterized by an early age of disability onset and early age of death in a subgroup of acute and progressive MS cases with meningeal TLSs [16,22,23]. Furthermore, the presence of TLSs in the meninges of MS cases was found to be linked to a “surface-in” gradient of subpial cortical neurodegeneration and glia activation, significantly higher in the outer cortical layers, close to the CSF surface, when compared to the inner ones [9], suggesting that the meningeal TLSs in MS may represent intrathecal sources of inflammatory stimuli able to orchestrate a chronic local inflammatory environment. These changes have also been confirmed in brain biopsies of early onset MS cases and acute MS post-mortem brains [19,23,24]. More recently, the presence of lymphoid-like structures in the forebrain meninges of post-mortem progressive MS cases was associated with increased spinal cord meningeal inflammation, white and grey demyelination, and axon loss in motor and sensory tracts [25]. All these data, supporting a key role for meningeal inflammation in both brain and spinal cord locations in MS patients, suggest that the meninges may represent intracerebral/spinal niches favoring lymphocyte accumulation and activity. This is possibly due to the early ingress of immune cells across a compromised BBB, contributing therefore to the MS-specific compartmentalized chronic inflammation within the CNS space.

## 3. Subpial Lesions

**Figure 2:**
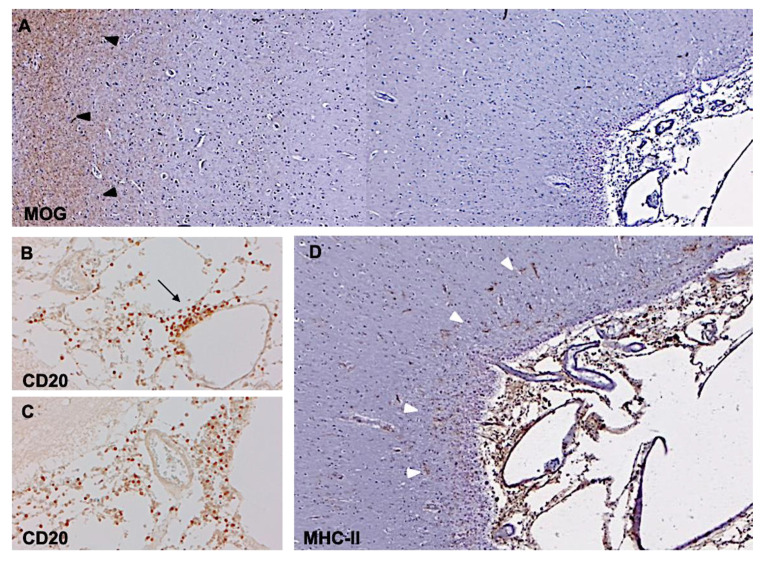
Demyelinating cortical lesion and meningeal inflammation in chronic progressive MS. Immunohistochemistry for myelin oligodendrocyte protein (MOG) shows extensive subpial demyelination involving the neocortical grey matter, contiguous to MOG-positive brain tissue (**A**, black arrowheads). The lesion is in close proximity to inflamed meninges containing an elevated number of CD20+ B cells, either accumulated (**B**, arrow) or diffused (**C**), around subarachnoid vessels. Substantial increased density of MHC-class II+ activated microglia can be observed in the most external cortical layers (white arrowheads) at the pia/CSF boundary (**D**) nearby inflamed meninges. Original magnifications: 50× (**A**), 100× (**D**), 200× (**B**,**C**).

In view of recent advances in imaging techniques, including brain MRI and PET, combined with the use of myelin immunohistochemistry in brain biopsies and post-mortem tissues, GM involvement in MS is again receiving considerable attention. The high prevalence of demyelination in GM areas in MS was originally detected by a study combining MRI and conventional histology in 12 post-mortem brains [26]. Based on the regional distribution of cortical lesions it was hypothesized that the observed pattern appeared to be causally related to the vascular anatomy, which resulted in the definition of seven distinct types of cortical lesions. Peterson et al. investigated cortical lesions in postmortem MS brains using myelin immunohistochemistry and proposed a classification system that distinguished three major cortical lesion types [27]. This scoring system is currently widely used for classification of cortical lesions in MS tissue and includes: (1) type 1 lesions, which are leukocortical areas of demyelination, extending across both white matter and gray matter but sparing the surface of the brain; (2) type 2 lesions, or “intracortical”, which are contained within the cerebral cortex grey matter and often occur around a blood vessel; and (3) type 3 lesions, or “subpial”, often affecting an extensive area of cortical demyelination (Figure 2A–D).

In one study, type-1 leukocortical lesions accounted for 15.5% of total lesions and affected 14.4% of the total cortical demyelinated area; type-2 intracortical lesions accounted for 16.5% of GM lesions and 1.2% of the demyelinated area; and type-3 subpial lesions were 60% of the total lesions and accounted for 67% of the total cortical demyelinated area. A further type, classified as type 4, was characterized by involvement of the whole cortical thickness and accounted for 8% of total lesions and 17% of demyelinated area [28]. Notably, when this classification system was applied to biopsy samples from patients with early stages of MS, 38% revealed clear evidence of cortical demyelination, with type-1 lesions accounting for half of the total number, followed by type-3 and type-2 plaques [19]. More importantly, cortical subpial demyelination has been identified as a peculiar and distinctive pattern occurring in a significant subpopulation of MS patients, particularly those with a long and progressive disease course [28]. The solidity of these data has been assessed and validated by several neuropathology studies [29,30].

A common appearance of type-3 lesions described in all neuropathology studies was that of long cortical ribbons of subpial demyelination, often affecting several adjacent gyri and stopping close to the WM boundary. Other type-3 lesions were wedge-shaped, with the base at the surface of the brain. Additionally, a combination of these patterns, with wedge-like lesion areas within bands of more superficial subpial demyelination, was often present [28].

Cortical grey matter lesions are characterized by a relative lack of lymphocyte infiltration, complement deposition or BBB disruption, as compared to WM lesions [31,32,33]. Moreover, cortical lesions are thought to be characterized by a dominant effector cell population of ramified microglia rather than macrophages [27]. The changes seen in GM appeared less severe compared to the WM and were not associated with increased cortical inflammation, astrogliosis, and complement deposition [33]. However, because of the anatomical proximity of the CSF space to the cortical GM, one must consider possible bi-directional immune cell trafficking between the subarachnoid space and the cortical parenchyma [34], or at least a degree of molecular exchange. Interstitial fluid in the subpial GM drains preferentially along perivascular (Virchow–Robin) channels to the CSF, while interstitial fluid in WM also spreads through the extracellular spaces between nerve fibers. In the WM of the brain, this fluid predominantly drains into the ventricles. These observations suggest that several molecules, in particular myelinotoxic substances, originating in the interstitial fluid could potentially circulate within Virchow–Robin spaces (resulting in perivenular demyelination) and the CSF (mediating subpial and periventricular demyelination), as will be extensively discussed below.

The diffusion of inflammatory molecules through Virchow–Robin spaces and pia mater may support the finding of a substantial “surface-in” gradient of subpial cortical neurodegeneration and glia activation, significantly higher in the outer cortical layers, close to the CSF surface, when compared to the inner ones close to the WM boundary [9], in particularly in MS patients with meningeal lymphoid-like structures. This cortical gradient has been replicated in vivo by surface-based analysis of ultra-high resolution MRI acquisition at 7 T [35] and was found to be more severe in progressive MS [36,37,38].

## 4. CSF Inflammation

Most of the MS-specific immunopathological features described above, in particular the intrathecal inflammation, are poorly revealed by conventional clinical and radiological tools. However, recent experimental evidence has suggested that CSF biomarkers may represent appropriate surrogates of MS intrathecal inflammation and may play a key role in diagnostic accuracy and monitoring of patients. Investigation of CSF has indeed regained attention in the last version of the McDonald diagnostic criteria, which include the presence of oligoclonal bands [39].

In addition to the presence of oligoclonal bands and increased intrathecal IgG synthesis that are indicative of chronic immune activation and represent a key diagnostic tool in MS, numerous molecules have been examined, including markers of tissue damage, such as the neurofilament light chain (NfL), and other biomarkers reflecting either pathophysiological processes (demyelination, inflammation, and repair), or for improving diagnosis and predicting disease progression and clinical outcome. The most desirable properties for biomarkers to be used in a clinical contest is that they provide: (1) a reliable and clear differentiation between MS and other demyelinating diseases; (2) an unambiguous distinction among relapsing and progressive MS courses; and (3) a predictable measure of treatment efficacy.

The study of CSF biomarkers of intrathecal inflammation is not only relevant for their diagnostic/prognostic role but also for elucidating molecular mechanisms of MS immunopathogenesis. Areas of application of CSF biomarkers in MS can be grouped as follows:

1. *Diagnostic*, including antibodies against aquaporin 4 and MOG, vascular cell adhesion molecules, glial fibrillary acidic protein (GFAP), complement components, cytokines (IL-6), and chemokines CXCL13);
2. *Prognostic*, including chitinase 3- like1 (CHI3L1), CXCL13, GFAP;
3. *Monitoring of therapy response and side effects*, including CXCL13, IL-6, IL-8CHI3L1, sCD21, sCD27 [40].

More recently, the integration of neuropathology with molecular and MRI methodologies has contributed to our understanding that specific proinflammatory cytokines (IFNγ, TNF, IL2, and IL22) and molecules related to sustained B-cell activity and lymphoid-neogenesis (CXCL13, CXCL10, LTα, IL6, IL10), characterize MS cases with higher levels of meningeal inflammation and GM demyelination at post-mortem analysis. The above CSF profile was also encountered in naive MS patients that at the time of diagnosis had increased cortical lesion volume and number [41], and, in addition, was strongly predictive of higher disease activity after 4 years of clinical and radiological longitudinal follow-up [42].

All these data corroborate the hypothesis that meningeal infiltrates may constitute the main source of CSF intrathecal inflammation [43], a view that has been recently confirmed by a new model of MS-like cortical pathology obtained by injecting lentiviral transfer vectors into the sagittal sulcus of Dark Agouti rats, in order to induce continuous expression of TNF + IFNγ. This experimental procedure resulted in meningeal inflammation and cortical GM pathology similar to that observed in MS patients [44].

At the same time, other studies suggested that several biomarkers, including IL12B, CD5, MIP1a, CXCL9, CCL11, and CCL20, highlight the independent yet complementary importance of T cells in disease pathogenesis [45]. Finally, iron homeostasis markers, such as sCD163 and hemoglobin, together with proteins of the coagulation cascade (fibrinogen) may also demonstrate the potential CSF signature of innate (monocyte/macrophage) activity at early disease stages [10].

## 5. Choroid Plexus Inflammation

The choroid plexus (CP) of the brain ventricles is a specialized structure composed of a connective stroma containing fenestrated capillaries encased by a layer of cuboidal epithelial cells that are linked by adherens junctions and apical tight junctions, and provide the anatomical substrate responsible for the blood–CSF barrier [46,47]. The CP epithelium is in direct continuity with ependymal cells, which in turn provide an immunological barrier between the CSF and the CNS, and also regulates bidirectional molecular trafficking, circulation, clearance, and metabolism of the CSF. In addition to CSF secretion, a function under the control of adrenergic and cholinergic innervation, the CP exerts a number of vital functions, encompassing maintenance of ion homeostasis, clearance of waste products, hormone production, and active transport of micronutrients and water-soluble vitamins [47] Moreover, the CP plays a pivotal role in regulating trafficking of immune cells from and to the CSF and brain parenchyma and in orchestrating an active immunosurveillance system. Under physiological conditions, the CP stroma is an immunologically active compartment densely populated by MHC class II-expressing M2-like macrophages, epiplexus Kolmer cells, and IL-10-producing myeloid dendritic cells, which extend projections into the ventricles for antigen uptake and presentation to stromal effector/memory CD4+ T cells, and additional cellular elements, such as NK cells and CD8+ effector/memory T cells [48]. Owing to its strategical location at the interface between blood and CSF, the CP has recently attracted particular interest as a fundamental player in CNS immunosurveillance and as a key culprit in acute and chronic neuroinflammation [49]. In parallel, attention is being devoted to the role of ependymal cells under physiological conditions and in neuroinflammatory disorders associated with ependymal dysfunction [50]. However, the role of CP in MS has been largely overlooked and unexplored compared to meningeal inflammation.

**Figure 3:**
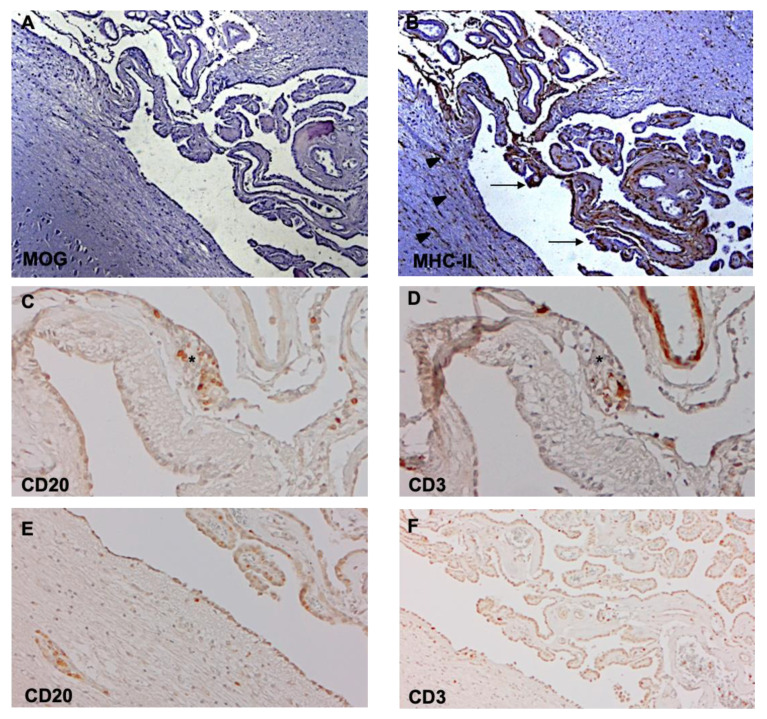
Periventricular demyelination and choroid plexus inflammation in chronic progressive MS. Immunohistochemistry for myelin oligodendrocyte protein (MOG) discloses subependymal demyelination (**A**) and an elevated number of MHC-class II+ activated microglia/macrophages (**B**) in the choroid plexus (arrows) and at the boundary ependyma/CSF (arrowheads). Elevated number of CD20+ B cells (**C**,**E**) and CD3+ T cells (**D**,**F**) are detected close to the tela choroidea (**C**,**D**), which separates the meninges (left) from the choroid plexus (right), and in the choroid plexus (**E**,**F**). Original magnifications: 100× (**A**–**F**).

In EAE, an experimental model for MS, neuroinflammation and demyelination are preceded by increased expression of ICAM-1, VCAM-1, and MAdCAM-1 by the CP epithelium. These changes are accompanied by leukocyte recruitment and focal necrosis of individual epithelial cells [51]. Moreover, recent studies show that the CP activates immune signaling in response to peripheral inflammation, thereby providing a compartmentalized niche for the homing of activated and proliferating CD4+ T cells [52]. While caution is necessary when translating experimental data to humans, a recent study shows an increased number of granulocytes and CD8+ T cells, but not B cells and plasma cells, in the CP stroma of subjects with progressive MS [53]. This is at variance with earlier reports showing an increased number of macrophages, dendritic cells, T cells, and CD138+ plasma cells in CP stroma in association with increased expression of VCAM-1 in endothelium capillaries [54]. Moreover, indirect evidence of CP inflammation has been provided by studies showing an increase in B cells, plasma cells, and memory and effector T cells in the CSF of patients with MS, a finding highly suggestive of their passage through the CP [55,56]. Recently, we also observed CP inflammation in association with periventricular deep GM demyelination in post-mortem brain tissues from subjects with chronic progressive MS (Magliozzi, unpublished) (Figure 3A–F).

The role of the choroid plexus and of the ependymal barrier warrants more attention in future studies of inflammatory neurological conditions, and specifically in demyelinating disorders to assess cellular trafficking (Figure 1D).

## 6. Conclusions

It is now clear that intrathecal inflammation is a characteristic of the clinical and pathological progression in MS. The compartmentalized immune response in the CSF-filled compartments of the brain, namely the ventricular and subarachnoid spaces, is closely associated with chronic demyelination and neurodegeneration, particularly in the cortical GM. Recent studies of cellular trafficking and molecular exchange between these compartments and the composition of the CSF has given rise to the hypothesis that a proinflammatory milieu can have damaging consequences to the tissues being bathed. Further studies are now required to identify the mechanisms by which intrathecal inflammation is promoted and maintained so that a rational approach can be developed for inhibiting the pathological progression.
